# Association of epilepsy with utilization and acute outcomes following cardiac surgery

**DOI:** 10.1371/journal.pone.0359534

**Published:** 2026-10-30

**Authors:** Konmal Ali, Syed Shaheer Ali, Troy Coaston, Sona Mahrokhi, Peyman Benharash

**Affiliations:** 1 Department of Surgery, Cardiovascular Outcomes Research Laboratories (CORELAB), University of California, Los Angeles, CA, United States of America; 2 Department of Surgery, Division of Cardiac Surgery, University of California, Los Angeles, CA, United States of America; Dow University of Health Sciences, PAKISTAN

## Abstract

**Purpose:**

Despite increased recognition of cardiovascular morbidity in patients with epilepsy, the influence of epilepsy on outcomes following cardiac operations remains poorly characterized.

**Methods:**

Adults (≥18 years) undergoing coronary artery bypass grafting (CABG), valve, or aortic procedures were identified in the 2016–2022 Nationwide Readmissions Database. Epilepsy was identified using validated International Classification of Diseases, 10th Revision (ICD-10) diagnosis codes (G40.x) present on a prior inpatient admission or as a non-primary diagnosis on the index admission; patients with epilepsy coded as the primary admitting diagnosis were excluded to avoid misclassifying acute presentations as preexisting disease. Entropy balancing across prespecified covariates (age, sex, Elixhauser Comorbidity Index, operation type, elective admission status, insurance type) was followed by mixed-effects regression with hospital-level random effects to assess the association of epilepsy with in-hospital mortality, postoperative complications, non-home discharge, and resource utilization including 30-day readmissions.

**Results:**

Of 1,932,935 adult patients undergoing cardiac operations, 27,325 (1.4%) had a preexisting diagnosis of epilepsy. On adjusted analysis, epilepsy was associated with significantly higher odds of in-hospital mortality (adjusted odds ratio [AOR]: 1.47; 95% confidence interval [CI]: 1.35–1.59), blood transfusion (AOR: 1.24; 95% CI: 1.20–1.29), infectious (AOR: 1.34; 95% CI: 1.27–1.40), and respiratory (AOR: 1.31; 95% CI: 1.26–1.37) complications. Epilepsy was also linked with prolonged hospitalization (β + 2.32 days; 95% CI: 2.12–2.47), greater costs (β + $9,121; 95% CI: 8,292−9,951), higher odds of non-home discharge (AOR: 2.01; 95% CI: 1.93–2.10), and 30-day non-elective readmission (AOR: 1.28; 95% CI: 1.21–1.34).

**Conclusions:**

Among patients undergoing cardiac operations, epilepsy was associated with increased risk of mortality, complications, and resource utilization. These findings demonstrate the need for enhanced perioperative strategies tailored to mitigate adverse events in this vulnerable population.

## Introduction

Patients with epilepsy are increasingly recognized to be at risk for cardiovascular events often not related to an acute seizure episode [1]. Coined Sudden Unexpected Death in Epilepsy (SUDEP), this phenomenon is thought to potentially stem from electrical disturbances in the heart increasing the risk of arrhythmias [2–4]. Beyond SUDEP, mounting evidence supports the concept of an “Epileptic Heart,” which is defined as muscle and coronary vascular damage attributable to repeated catecholamine storms and hypoxia [3]. Postmortem studies of patients with epilepsy have uncovered pathologic changes to the heart including atherosclerosis, myocardial fiber degeneration and ventricular dilation [3,5]. Epidemiological data further underscore this association, demonstrating a 21% incidence of heart disease in adults with epilepsy compared to approximately 12% in others [5]. With improved life expectancy and cardiodiagnostics, an increasing number of patients with epilepsy are anticipated to require cardiac operations [6].

Cardiac surgery poses unique challenges to brain function through various mechanisms including arterial plaque embolization, ischemia-reperfusion injury and introduction of pharmacologic agents which may reduce the seizure threshold [5,7]. While literature has characterized neurologic injury and seizures arising from operations, the influence of preexisting epilepsy on postoperative outcomes has not been examined at a national level. In the absence of adequate characterization of postoperative outcomes, the diagnosis of epilepsy may not trigger adequate consideration and preoperative optimization [8]. Moreover, a comprehensive understanding of such outcomes will facilitate shared and informed decision making regarding the choice of intervention.

In the present work, we characterized the association of epilepsy with acute outcomes of cardiac operations in a national cohort. We hypothesized epilepsy to be associated with greater odds of in-hospital mortality, postoperative complications, and increased resource utilization including length of stay, costs, and 30-day readmissions.

## Methods

In this retrospective analysis all adult (≥18 years) hospitalizations entailing coronary artery bypass grafting (CABG), all valve or aortic procedures were tabulated from the 2016−2022 Nationwide Readmissions Database (NRD) using previously published International Classification of Diseases 10th Revision (ICD-10) procedural codes [9]. This 7-year study period was selected to coincide with the first complete year of ICD-10 coded data through the most recently available year at the time of data access, thereby maximizing both coding consistency and contemporary relevance. Operations were classified as CABG regardless of isolated or combination status, valve comprising isolated as well as multivalve procedures, and aortic operations with operation type included as a covariate in all adjusted models. The NRD is the largest-all payer readmissions database in the United States and provides accurate estimates for nearly 60% of all hospitalizations [9]. Unique patient identifiers allow for tracking of readmissions across hospitals within each state and calendar year. Entries missing key data elements (4.7%) or a diagnosis of endocarditis, concomitant ventricular assist device placement or heart transplantation, were excluded (1.3% Fig 1).

**Fig 1:**
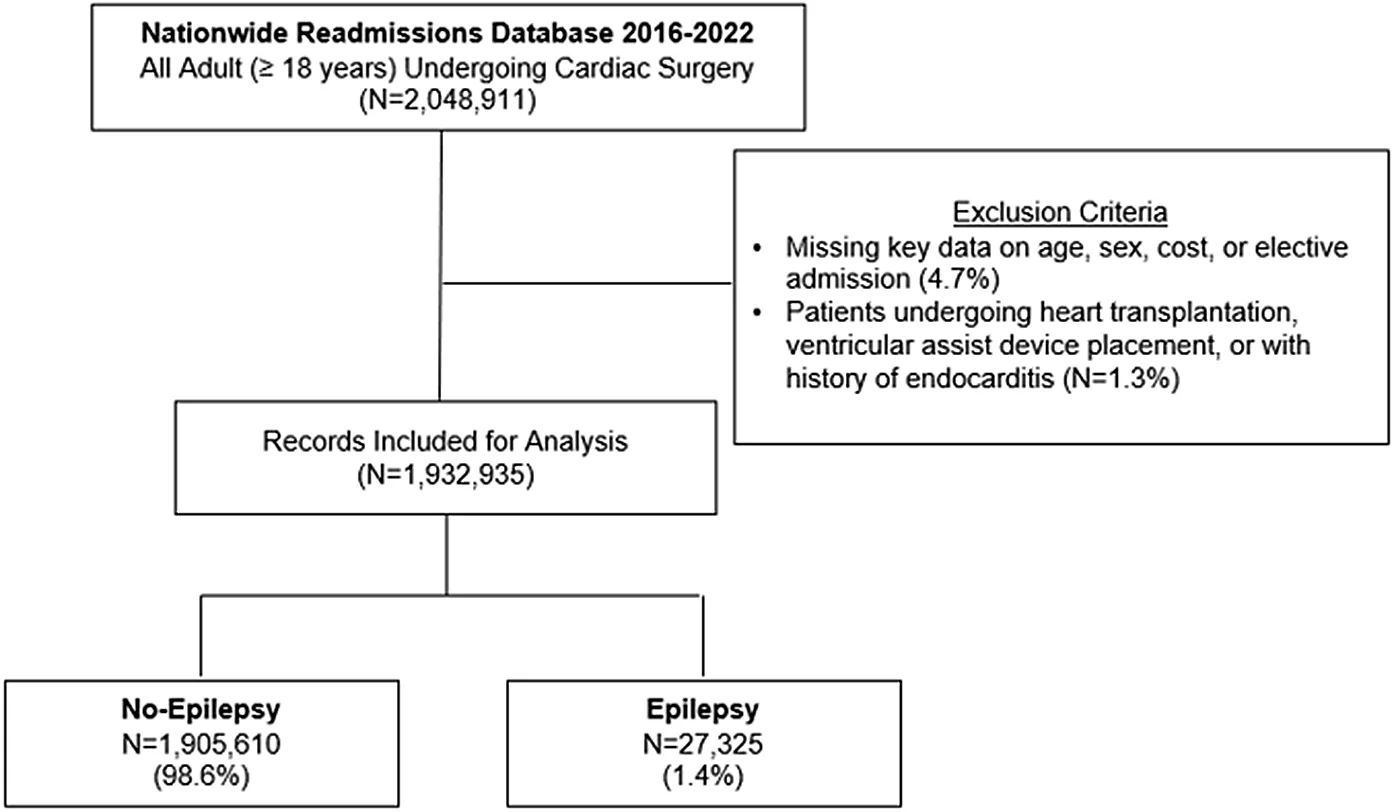
Study Flow Diagram of Survey-Weighted Estimates depicting the initial cohort, applied exclusion criteria, and final analytic subgroups.

The NRD Data Dictionary was utilized to define procedure, patient, and hospital characteristics [10]. The presence of the diagnosis for epilepsy was ascertained using previously validated ICD-10 diagnosis code G40 present on or before the index admission [11]. Additionally, patients with new-onset seizures or epilepsy-related readmissions identified only after the index hospitalization were not included in this group. The burden of chronic conditions was quantified using the van Walraven modification of the Elixhauser Comorbidity Index, a composite score of 30 chronic conditions. Complications were categorized as cardiac (cardiac tamponade, myocardial infarction, ventricular fibrillation, ventricular tachycardia), infectious (wound infection, postoperative seroma, cellulitis, superficial/deep site infection), respiratory (acute respiratory distress syndrome, pneumonia, pneumothorax, prolonged ventilation, respiratory failure), neurologic (intracerebral hemorrhage, acute ischemia, stroke), renal (acute kidney injury), or thromboembolic (deep vein thrombosis, pulmonary embolism, S1 Table). Non-home discharge was characterized as a disposition to acute care, short-term, and skilled nursing facilities. Hospitalization costs were calculated via the application of center-specific cost-to-charge ratios to overall charges followed by inflation adjustment to the 2021 Personal Healthcare Price Index [12].

The primary outcomes of interest were adjusted in-hospital mortality and the development of postoperative complications during the index hospitalization. Adjusted Length of stay (LOS), costs, non-home discharge, and non-elective readmissions within 30 days of index discharge, were secondarily evaluated.

Categorical variables are reported as proportions (%) and continuous variables as medians with interquartile range (IQR). The significance of intergroup differences at the patient level was evaluated using the absolute standardized mean difference (SMD). Bivariate comparisons were performed using Pearson’s Χ^2^ and Mann-Whitney U tests, as appropriate. To account for baseline intergroup differences, entropy balancing was first utilized to generate balanced subpopulations while preserving the entire sample size for analysis [13]. Covariates incorporated in entropy balancing included age, sex, Elixhauser Comorbidity Index, elective admission status, operation type, insurance type, hospital volume, and hospital teaching status. We subsequently constructed mixed regression models with hospital identifier incorporated as a random effect to account for hospital-level clustering, while all aforementioned covariates were specified as fixed effects, achieving a doubly robust analysis [14]. Covariate selection was then guided by elastic net regularization with an alpha value of 0.5 and lambda selected via 10-fold cross-validation to mitigate the probability of overfitting, collinearity, and bias [15]. Model performance was assessed using receiver operating characteristics (C-statistic), calibration plots, and coefficients of determination (R^2^), as appropriate. Regression estimates are shown as adjusted odds ratios (AOR) for logistic and beta-coefficients (β) for linear models, both with 95% confidence intervals (95% CI). The Nelson-Aalen method was used to estimate the cumulative hazard of all-cause non-elective readmission within 30 days of discharge. Patients who died during the index hospitalization were not included in readmission analyses. Statistical significance was defined as a P-value < 0.05 or SMD ≥ 0.10. All statistical analyses were performed using Stata 18.1 (StataCorp, College Station, TX). This study was conducted and reported in accordance with the Strengthening the Reporting of Observational Studies in Epidemiology (STROBE) guidelines. Given the deidentified nature of the data source, this study was exempt from full review by the Institutional Review Board at the University of California, Los Angeles (IRB #17–001112). Data were accessed on November 2^nd^, 2025. Data utilized in this study were derived from the Nationwide Readmissions Database (NRD), which is publicly available upon purchase through the Healthcare Cost and Utilization Project (HCUP).

## Results

Of an estimated 1,932,935 adult patients undergoing cardiac operations, 27,325 (1.4%) had a preexisting diagnosis of epilepsy with detailed characteristics shown in Table 1.

**Table 1:** Patient, and hospital characteristics of patients undergoing cardiac surgery with and without epilepsy.

	*No-Epilepsy* (n = 1,905,610)	*Epilepsy* (n = 27,325)	*SMD*
Age (years [IQR])	67 [59-73]	64 [56-72]	0.21
Female (%)	28.6	35.9	0.15
Elixhauser Index (median [IQR])	5 [3–6]	6 [5–8]	0.70
Elective (%)	54.8	49.7	0.10
*Operation Type (%)*			0.24
Aortic	8.0	11.4	
CABG	72.2	60.5	
Valve	35.5	45.4	
*Community income percentile (%)*			0.12
>75^th^	20.1	17.0	
51-75^th^	25.6	23.1	
26-50^th^	28.3	29.1	
0-25^th^	26.0	30.9	
*Insurance coverage (%)*			0.19
Private	31.4	22.2	
Medicare	55.1	58.7	
Medicaid	7.9	13.7	
Other Payer	5.6	5.5	
*Comorbidities (%)*			
Chronic pulmonary disease	17.4	20.6	0.08
Congestive heart failure	38.7	43.3	0.09
Diabetes	36.0	28.0	0.17
Hypertension	76.6	69.4	0.16
Liver disease	3.9	4.5	0.04
Obesity	29.9	25.2	0.10
*Hospital teaching status (%)*			0.02
Non-Metropolitan	2.1	2.1	
Metropolitan Non-teaching	13.3	12.4	
Metropolitan Teaching	84.5	85.5	

IQR: Interquartile Range; SMD: Standardized Mean Difference; CABG: Coronary Artery Bypass Grafting.

Patients with epilepsy were younger (64 [56–72] vs 67 [59–73] years, SMD = 0.21), more commonly female (35.9 vs 28.6%, SMD = 0.15), and had a greater median Elixhauser Comorbidity Index (6 [5–8] vs 5 [3–6], SMD = 0.70). Individuals with epilepsy were more frequently classified into the lowest income quartile (30.9 vs 26.6%, SMD = 0.12) and insured by Medicaid (13.7 vs 7.9%, SMD = 0.19). Furthermore, those with epilepsy more commonly underwent valve operations (45.4 vs 35.5%, SMD = 0.24) and aortic procedures (11.4 vs 8.0%, SMD = 0.24).

On crude analysis, patients with epilepsy faced higher rates of mortality (4.7 vs 2.9, SMD = 0.10, Table 2).

**Table 2:** Unadjusted outcomes of patients undergoing cardiac surgery with and without epilepsy.

	*No-Epilepsy* (n = 1,905,610)	*Epilepsy* (n = 27,325)	*SMD*
**Clinical outcomes**			
In-Hospital Mortality (%)	2.9	4.7	0.10
Additional complications (%)			
Blood Transfusion	19.4	24.5	0.12
Cardiac	30.6	30.6	0.01
Infectious	9.7	15.4	0.17
Renal	20.1	22.1	0.05
Respiratory	12.9	17.9	0.14
Thromboembolic	1.0	1.6	0.05
			
**Resource utilization**			
Costs ($1000s, median [IQR])	52.0 [38.3-74.2]	61.3 [43.4-91.7]	0.23
Length of Stay (days, median [IQR])	8 [6–12]	10 [7–17]	0.29
30-day readmission (%)	10.0	13.7	0.11
Non-Home Discharge (%)	16.6	27.8	0.27

IQR: Interquartile Range; SMD: Standardized Mean Difference.

Additionally, those experiencing epilepsy more frequently required blood transfusion (24.5 vs 19.4%, SMD = 0.12) as well as had greater rates of infectious (15.4 vs 9.7%, SMD = 0.17) and respiratory (17.9 vs 12.9%, SMD = 0.14) complications. Notably, patients with epilepsy had a longer LOS (10 [7–17] vs 8 [6–12] days, SMD = 0.29), more often discharged to non-home settings (27.8 vs 16.6, SMD = 0.27), and demonstrated increased rates of 30-day non-elective readmission (13.7 vs 10.0%, SMD = 0.11), compared to others. Furthermore, those with epilepsy had greater hospitalization costs ($61,312 [43,400−91,726] vs $52,144 [38,315−74,241]).

Adjusted outcomes are shown in Table 3.

**Table 3:** Risk-Adjusted outcomes of patients undergoing cardiac operations with and without epilepsy. Estimates from dichotomous outcomes are reported as AORs and continuous outcomes as β-coefficients.

	*Estimate (AOR/β)*	*95% CI*
**Clinical outcomes**		
In-hospital mortality	1.47	1.35-1.59
Additional complications		
Blood transfusion	1.24	1.20-1.29
Cardiac	1.02	0.98-1.06
Infectious	1.34	1.27-1.40
Renal	1.04	1.00-1.09
Respiratory	1.31	1.26-1.37
Thromboembolic	0.94	0.82-1.08
		
**Resource utilization**		
Costs (USD $1000s, β)	+9.12	8.29-9.95
Length of stay (days, β)	+2.32	2.12-2.47
30-day readmission (AOR)	1.28	1.21-1.34
Non-home discharge (AOR)	2.01	1.93-2.10

AOR, Adjusted Odds Ratio; CI, Confidence Interval.

Following entropy balancing and multivariable risk adjustment (Fig 2), epilepsy was associated with significantly increased odds of mortality by a relative risk increase of 47% (AOR 1.47, 95% CI 1.35–1.59, C-Statistic 0.79).

**Fig 2:**
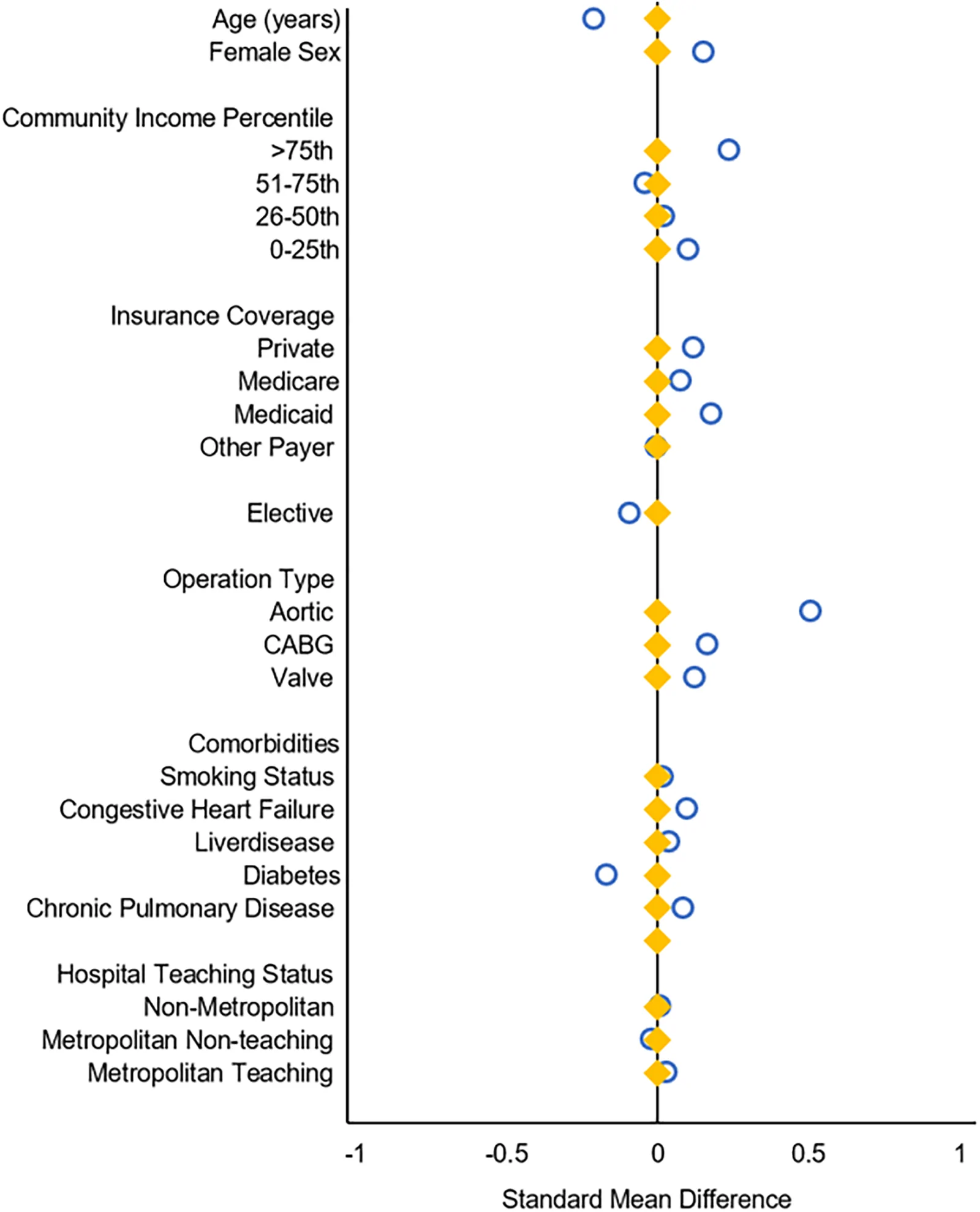
Covariate balance plot demonstrating standardized mean differences (SMDs) before (open circles) and after (closed diamonds) entropy balancing among individuals with and without a diagnosis of epilepsy. Post-balancing SMDs < 0.10 indicate adequate covariate balance.

Epilepsy was linked with greater odds of blood transfusion (AOR 1.24, 95% CI 1.20–1.29), infectious (AOR 1.34, 95% CI 1.27–1.40), and respiratory (AOR 1.31, 95% CI 1.26–1.37, Fig 3) complications.

**Fig 3:**
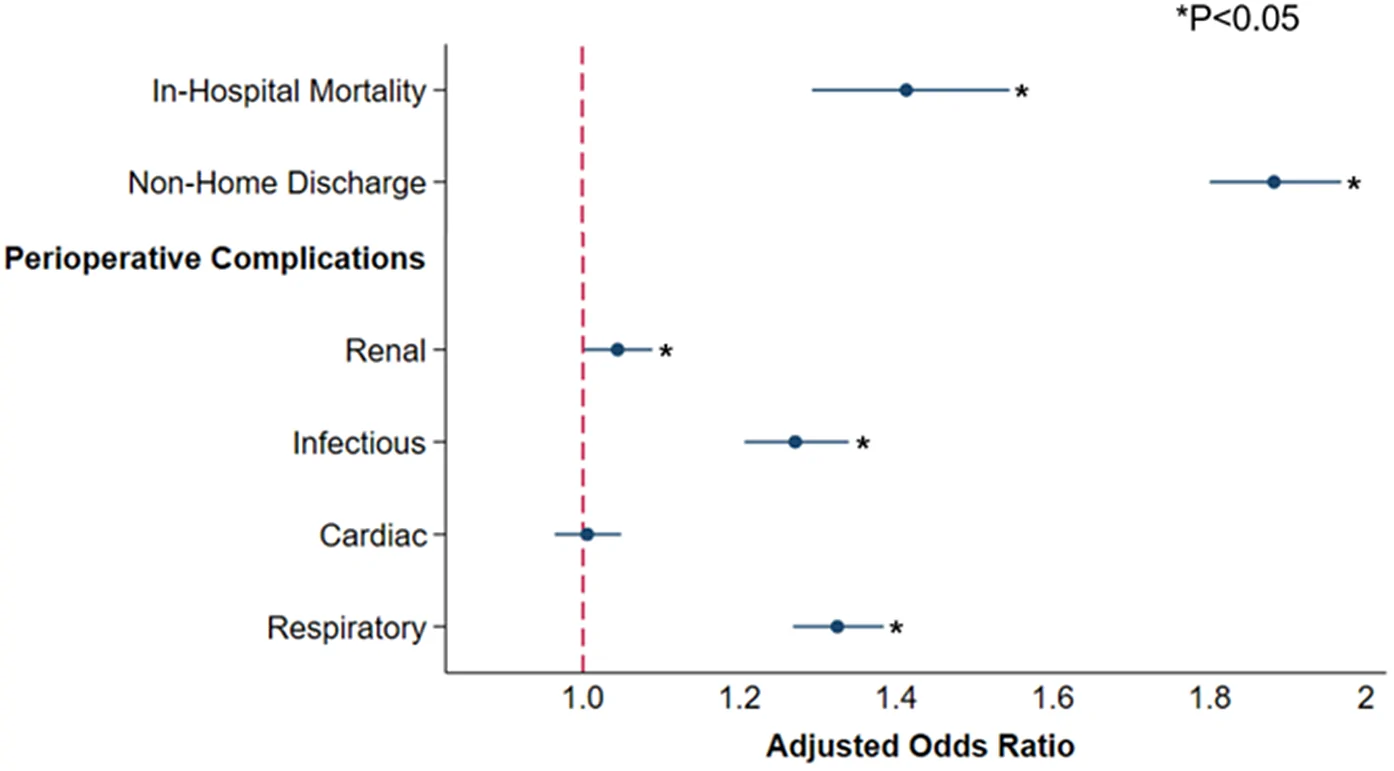
Forest plot depicting risk-adjusted association of epilepsy with perioperative outcomes following cardiac surgery. Following comprehensive risk-adjustment, epilepsy was associated with increased in-hospital mortality and postoperative complications. Estimates are reported as adjusted odds ratios (AOR) with 95% confidence intervals. Reference group: No-Epilepsy; *asterisks denote estimates significant at P < 0.05.*

Epilepsy was associated with greater hospitalization costs (β + $9,121, 95% CI 8,292−9,951), longer LOS (β + 2.32 days, 95% CI 2.12–2.47), and higher odds of non-home discharge (AOR 2.01, 95% CI 1.93–2.10). Notably, epilepsy was associated with increased odds of non-elective readmission within 30 days with a relative risk increase of 28% (AOR 1.28, 95% CI 1.21–1.34). Further analysis revealed a significant rise in risk of readmission among those with epilepsy as shown in Fig 4.

**Fig 4:**
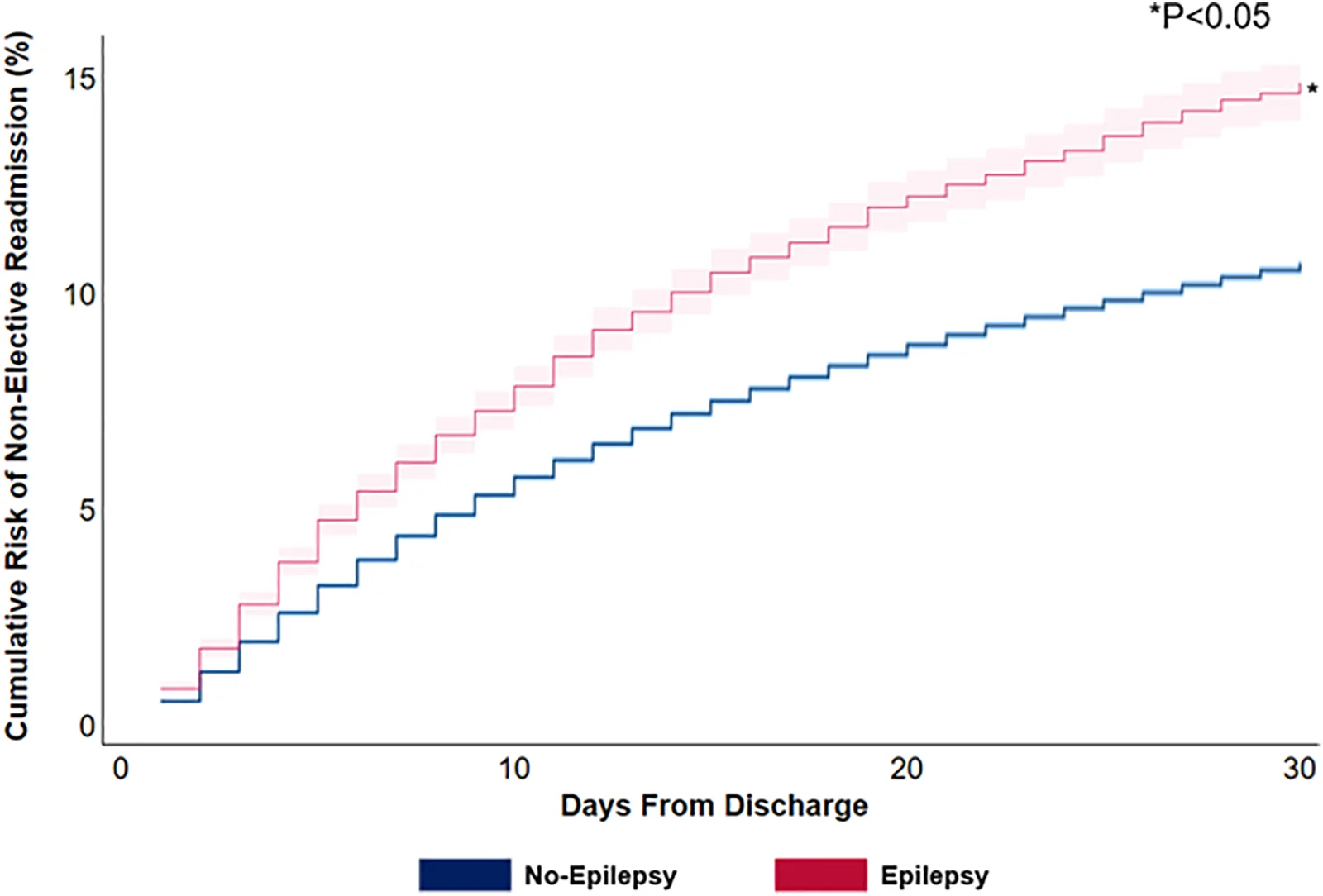
Nelson-Aalen cumulative hazard curves depicting the risk of non-elective readmission within 30 days of discharge among patients with and without epilepsy. The epilepsy cohort demonstrates a significantly greater cumulative risk throughout the follow-up period *(P < 0.05).*

Moreover, epilepsy continued to be associated with higher adjusted rates of mortality with increasing age (Fig 5).

**Fig 5:**
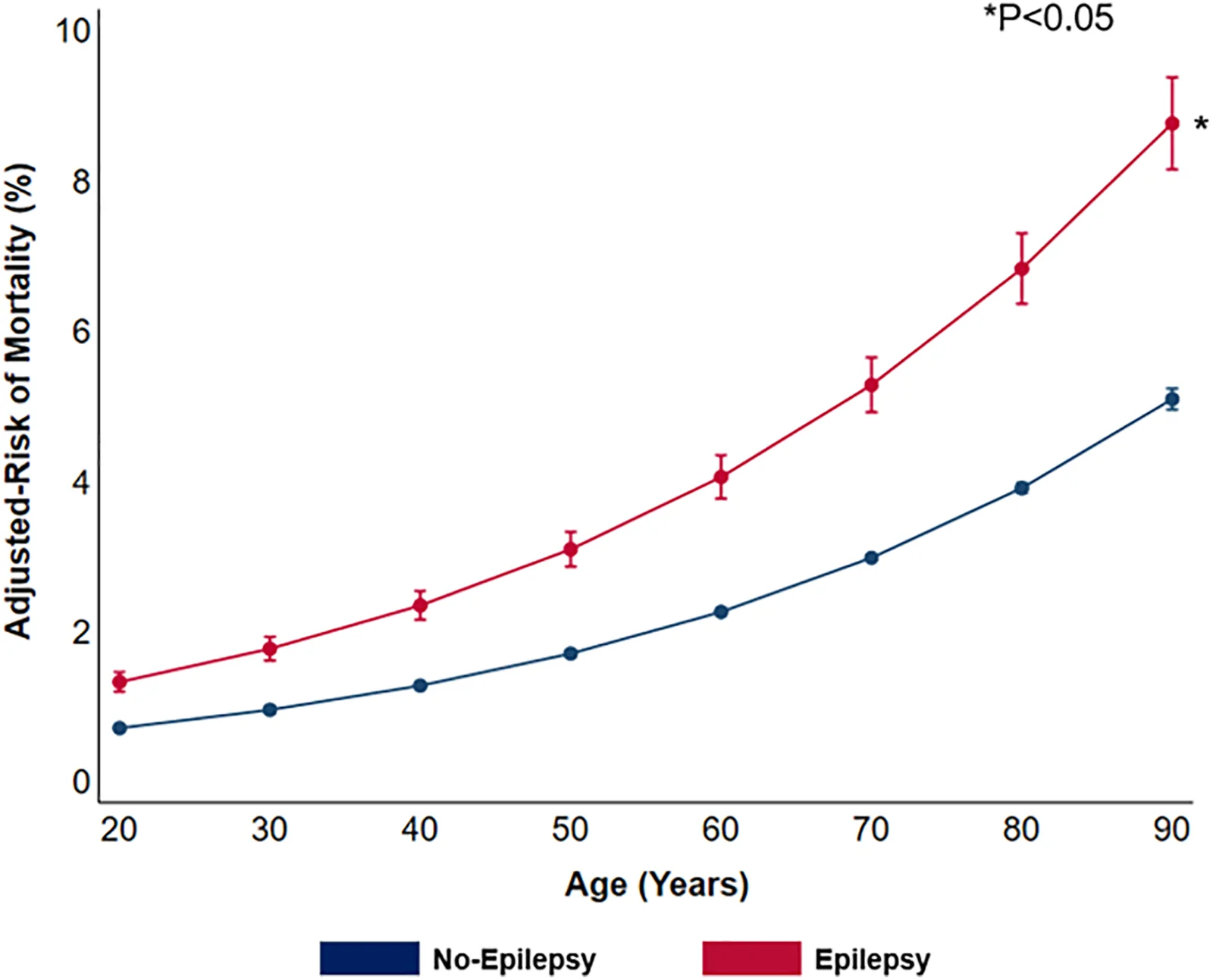
Risk-adjusted mortality rates stratified by age at admission among patients with and without epilepsy. Error bars represent 95% confidence intervals. The epilepsy cohort demonstrates consistently greater adjusted mortality across all age groups *(P < 0.05).*

## Discussion

The existing cardiac surgery literature has been limited to neurologic injury and seizures stemming from cardiac operations rather than preexisting epilepsy. In the present national analysis, we examined the link between epilepsy and short-term outcomes of common cardiac operations and made several important observations. We observed that patients with epilepsy undergoing cardiac operations were associated with significantly elevated risks across multiple outcome domains with a 47% relative increase in the odds of death and substantially higher rates of infectious, respiratory, and renal complications. Moreover, patients with epilepsy face increased markers of resource utilization and rates of non-home discharge as well as unplanned rehospitalization. Several of these findings warrant further discussion.

In the present work, 1.4% of cardiac surgical candidates had a preexisting diagnosis of a seizure disorder. Importantly, the observed association between epilepsy and mortality remained pronounced even among older patients, where frailty and advanced age would be expected to dominate the risk profile [16–19]. While data could not be extracted from our dataset, the higher rate of complications, particularly the 34% increased odds of infectious complications and 31% increased odds of respiratory complications, may reflect seizure-related physiologic stress, medication effects on immune function, and the potential for aspiration events in the perioperative period, though direct evidence in such populations remains limited [20,21]. Previous studies have demonstrated antiepileptic drugs can impair immune function and wound healing, which may partially explain the increased infectious complications observed in our cohort [22,23]. Clinicians should consider enhanced preoperative optimization strategies for epilepsy patients, including neurologic consultation for seizure management, careful medication adjustment to avoid drug interactions potentially lowering seizure threshold, and possibly introduce monitoring protocols for early detection and management of complications [24,25].

The observed increase in the risk of various complications among epilepsy patients may, at least in part, be explained by pathophysiologic mechanisms extending beyond traditional cardiovascular risk factors [26]. The concept of the “Epileptic Heart” provides a framework for understanding how chronic seizure activity may predispose patients to both cardiac pathology and increased surgical vulnerability through repeated catecholamine storms, episodes of hypoxia, and dysregulation of the autonomic nervous system [27,28]. It is plausible that cardiopulmonary bypass-induced inflammatory responses exacerbate preexisting brain inflammation, that anesthetic and clot-preventing agents lower the seizure threshold, and that the inherent stress of surgery triggers seizures [29,30]. Moreover, Fountain and colleagues have shown seizure activity to result in prolonged immunosuppression and altered respiratory mechanics, potentially explaining the increased susceptibility to these complications [31]. Taken together, the significantly increased risk of adverse events should be taken into consideration when choosing therapeutic strategies and discussed with patients and their families.

After multivariable risk adjustment, epilepsy was linked with increased duration of hospitalization and episodic costs. This finding may be attributable to the higher incidence of complications in the postoperative period among such patients. Notably, nearly 30% of patients with epilepsy required non-home discharge while ~14% were rehospitalized within 30 days of discharge [32]. Such findings have implications towards both value of care and patient-centered outcomes. With the adoption of interventional procedures in the management of coronary and valvular heart disease, physicians and patients must choose among therapies aligning with patients’ wishes. Increased hospitalization costs and non-home discharge may further burden patients during the recovery period and adversely impact their sense of well-being and autonomy. Thus, a realistic understanding of postoperative outcomes should better guide the choice of modality and expectations. The significant risk of readmissions further compounds the economic burden while reducing hospital free days, an emerging metric of patient satisfaction. Our findings suggest the relative inadequacy of current discharge planning and transitional care protocols in this high-risk population [33]. Development of epilepsy-specific perioperative care pathways integrating neurologic and cardiovascular care following discharge may enhance the care of such patients and paradoxically reduce expenditures. Similar integrated care models have shown promise in other high-risk populations, such as patients with chronic kidney disease undergoing cardiac surgery [34]. Future research should focus on identifying modifiable risk factors within the epilepsy population, evaluating the effectiveness of targeted interventions and developing risk prediction models incorporating epilepsy [35,36].

The present study is subject to several limitations inherent to administrative database research. As with all large administrative databases, the NRD relies on hospital billing and diagnostic coding practices, which may introduce variability in case identification and outcome ascertainment. Furthermore, the NRD does not capture granular clinical information such as epilepsy severity, duration, or frequency of seizures, antiepileptic medication regimens, laboratory values, operative complexity, or imaging findings, all of which may influence both surgical decision-making and postoperative course. The inability to differentiate between active and well-controlled epilepsy represents a meaningful limitation, as disease severity likely modulates perioperative risk in ways that cannot be fully characterized from administrative data alone. Additionally, the NRD does not allow tracking of patients across state lines or beyond a single calendar year, which may result in undercounting of readmissions and limit longer-term follow-up. Given the small sample size of the epilepsy cohort, subgroup analyses were not conducted to avoid false associations. However, this may be an adequate area of future work among institutional studies. Despite these limitations, the present study leverages the largest all-payer readmissions database in the United States to provide the first national characterization of cardiac surgical outcomes among patients with preexisting epilepsy, offering findings that are broadly generalizable and clinically actionable.

In summary, we found epilepsy to be associated with greater odds of in-hospital mortality, postoperative complications, and 30-day non-elective readmissions. We further noted epilepsy to be linked with greater hospitalization costs and length of stay. With the anticipated rise in the prevalence of epilepsy and heart disease in the aging population, these findings suggest that further investigation into targeted perioperative strategies for this vulnerable population is warranted.

## Supporting information

S1 Table ICD-10 codes for medical and surgical complications. (DOCX)

## Abbreviations

AOR: Adjusted Odds Ratio
CABG: Coronary Artery Bypass Grafting
CI: Confidence Interval
IQR: Interquartile Range
LOS: Length of Stay
NRD: Nationwide Readmissions Database
SMD: Standardized Mean Difference
SUDEP: Sudden Unexpected Death in Epilepsy
