# Supplementary material for: Association of epilepsy with utilization and acute outcomes following cardiac surgery
Source: PLoS One. 2026 Oct 30;21(10):e0359534. doi: 10.1371/journal.pone.0359534 (PMC13722181; doi:10.1371/journal.pone.0359534)
Supplement: Supplementary file 1 [file pone.0359534.s001.docx]

**Supplemental Table 1.**

ICD-10 Codes for Medical and Surgical Complications

|  | ***ICD-10 Codes*** |
| --- | --- |
| **Complications** |  |
|  |  |
| Cerebrovascular |  |
| Intracranial hemorrhage | I62.1, I62.0, I61.9, I62.9, I60.9 |
| Stroke | G97.81, G97.82, I97.811, I97.821 |
| Transient ischemic attack | G45.4, I63.019, I63.119, I63.139, I63.20, I63.219, I63.22, I63.239, I63.30, I63.40, I63.50, I63.59, I67.2, I67.5, I67.7, I67.81, I67.82, I67.89, I67.9 |
|  |  |
| Cardiac |  |
| Arrest | I46.2, I46.8, I46.9 |
| Ventricular fibrillation | I49.01 |
| Ventricular tachycardia | I47.2 |
| Tamponade | I31.4 |
| Myocardial infarction | I21 |
|  |  |
| Respiratory |  |
| ARDS | J80, R06.03 |
| Pneumonia | J12.0, J12.1, J12.2, J12.81, J12.89, J12.9, J13, J14, J15.0, J15.1, J15.20, J15.211, J15.212, J15.29, J15.3, J15.4, J15.5, J15.6, J15.7, J15.8, J15.9, J16.0, J16.8, J18.0, J18.1, J18.9, J95.851, J95.89 |
| Pneumothorax | J95.811 |
| Prolonged mechanical  Ventilation > 48 hours | 5A1855Z |
| Respiratory failure | J95.821, J95.822, J96.00, J96.20, J96.90 |
|  |  |
| Renal |  |
| Acute renal failure requiring  dialysis | N18.4, N18.5, N18.6, N17.0, N17.2, N17.8, N17.9, N17, V54.11, Z99.2 |
|  |  |
| Infectious |  |
| Sepsis | A40.3, A40.9, A41.01, A41.02, A41.1, A41.2, A41.3, A41.4, A41.50, A41.9, K68.11, R65.20, T80.211A, T80.212A, T80.219A, T80.22XA, T80.29XA, T81.4XXA |
| Surgical site infection | T81.31XA, T81.32XA, T81.4XXA, K68.11 |
|  |  |
| Thrombotic |  |
| Deep vein thrombosis | I80.10, I80.209, I80.219, I80.3, I82.429, I82.439, I82.449, I82.499, I82.4Y9, I82.890, I82.91, I82.A19, I82.B19, I82.C19 |
| Pulmonary embolism | 415.1, 415.11, 415.12, 415.19, I26.90, I26.99, T800XXA, T81718A, T8172XA, T82817A, T82818A |
|  |  |
| Intraoperative |  |
| Accidental puncture | D78.11, D78.12, E36.11, E36.12, G97.48, G97.49, H59.219, H5.9229, H95.31, H95.32, I97.51, I97.52, J95.71, J95.72, K91.71, K91.72, L76.11, L76.12, M96.820, M96.821, N99.71, N99.72, T888XXA |
| Hemorrhage | D7801, D7802, D7821, D7822, E3601, E36.02, E89.810, E89.811, G97.31, G97.32, G97.51, G97.52, H59.111, H59.112, H59.113, H59.119, H59.313, H59.319, H59.321, H59.322, H59.323, H593.29, H95.21, H95.22, H95.41, H95.42, I97.410, I97.411, I97.418, I97.42, I97.610, I97.611, I97.618, I97.620, J95.61, J95.62, J95.830, J95.831, K91.61, K91.62, K91.840, K91.841, L76.01, L76.02, L76.21, L76.22, M96.810, M96.811, M96.830, M96.831, N99.61, N99.62, N99.820, N99.821 |
| Blood transfusion | 30233H0, 30233N0, 30243H0, 30243N0, 30253H0, 30253N0, 30263H0, 30263N0, 30233H1, 30243H1, 30253H1, 30263H1, 30233H0, 30233N0, 30233W0, 30243H0, 30243N0, 30243W0, 30253H0, 30253N0, 30253W0, 30233H0, 30233N0, 30233W0, 30243H0, 30263H0, 30263N0, 30263W0, 30233H1, 30243H1, 30253H1, 30263H1, 30233N1, 30233P1, 30243N1, 30243P1, 30253N1, 30253P1, 30263N1, 30263P1, 30233R1, 30243R1, 30253R1, 30263R1, 30233T1, 30233V1, 30233W1, 30243T1, 30243V1, 30243W1, 30253T1, 30253V1, 30253W1, 30263T1, 30263V1, 30263W1, 30233J1, 30233K1, 30233L1, 30233M1, 30243J1, 30243K1, 30243K1, 30243M1, 30253J1, 30253K1, 30253L1, 30253M1, 30263J1, 30263K1, 30263L1, 30263M1, 30233Q1, 30243Q1, 30253Q1, 30263Q1 |
